# Expression of genes of the aflatoxin biosynthetic pathway in *Aspergillus flavus* isolates from keratitis

**Published:** 2011-11-11

**Authors:** George Leema, Duen-Suey Chou, Christadoss A. Nelson Jesudasan, Pitchairaj Geraldine, Philip A. Thomas

**Affiliations:** 1 Department of Animal Science, School of Life Sciences, Bharathidasan University, Tamil Nadu, India; 2 Department of Pharmacology, School of Medicine, College of Medicine, Taipei Medical University, Taipei, Taiwan; 3 Institute of Ophthalmology, Joseph Eye Hospital, Tamil Nadu, India

## Abstract

**Purpose:**

To document transcriptional activation (expression) of key aflatoxin biosynthetic pathway genes in corneal isolates of *Aspergillus flavus*.

**Methods:**

The expression of certain regulatory (aflatoxin regulatory [*aflR*] and aflatoxin J [*aflJ*]) and structural (polyketide synthase acetate [*pksA*] and norsolonic acid-1 [*nor-1*]) genes in four corneal *A*. *flavus* isolates was evaluated by reverse transcription PCR. The aflatoxin-producing potential of each strain was determined by thin-layer chromatography and quantified by spectrophotometry. Four environmental isolates were used for comparison. The mean expression levels of these genes were compared in the corneal and environmental *A*. *flavus* isolates. In addition, the mean expression levels were also correlated with the aflatoxin production levels.

**Results:**

All isolates expressed *aflJ*, *nor-1*, and *pksA*, while all but one expressed *aflR*. Overall, significantly higher mean expression levels occurred in aflatoxigenic than in non-aflatoxigenic corneal isolates. A significant positive correlation was noted between the mean expression level of *aflR* and the quantum of aflatoxin production by the corneal isolates. Essentially similar patterns of expression of these genes were noted in four environmental *A*. *flavus* isolates used for comparison.

**Conclusions:**

For the first time, isolates of *A. flavus* from human keratitis patients have been shown to express regulatory and structural aflatoxin biosynthetic pathway genes. Further studies are needed to clarify the precise influence of the corneal microenvironment on expression of these genes and aflatoxin production by *A. flavus* infecting the cornea.

## Introduction

*Aspergillus flavus* is an important cause of keratitis [1], and is reported in some studies to be the most frequent *Aspergillus* species causing keratitis [2-4]. Recent experimental studies on pathogenesis of fungal keratitis have tended to focus on *Aspergillus fumigatus* [5-8], even though *A. flavus* is known to produce potent mycotoxins, the aflatoxins, that are potentially harmful to humans and animals [9]. Moreover, the aflatoxin biosynthetic pathway is well characterized, with 25 genes known to be involved [10-12]. The expression of these genes has been studied in isolates of *A. flavus* from the field [13], but not in those from corneal sources, including the eye.

We have previously reported that aflatoxin B1(AFB1) was produced by eight of 10 strains of *A. flavus* isolated from corneal material of patients with keratitis but by only four of 10 strains of *A. flavus* isolated from the environment [14]. Since conidia in the environment are the major source of inoculum for *Aspergillus* species (including *A. flavus*) causing opportunistic infections in plants, animals and humans [15], roughly equal proportions of the corneal isolates and the environmental isolates should have exhibited aflatoxin-producing potential, whereas the actual difference was twofold. We speculated that this difference possibly arose from increased transcriptional activation (expression) of genes involved in aflatoxin biosynthesis in corneal isolates of *A. flavus*. Hence, in the present investigation, reverse transcription-PCR (RT–PCR) was used to study expression of two regulatory genes, aflatoxin regulatory (*aflR*) and aflatoxin J (*aflJ*), and two structural genes, norsolonic acid-1 (*nor-1*) and polyketide synthase acetate (*pksA*), of the aflatoxin biosynthetic pathway in four of the *A. flavus* corneal isolates used in our previous study; a possible relationship between expression of these genes and quantum of aflatoxin production by the isolates was also sought.

## Methods

### Fungal isolates

A detailed description of a collection of 10 corneal and 10 environmental isolates of *A. flavus* has been provided in a previous publication [14]. Two corneal isolates (C1 and C4 [GenBank HM_003455 and HM_003474, respectively]) that produced aflatoxin (aflatoxigenic isolates) and two corneal isolates (C2 and C7 [GenBank HM_003456 and HM_003459, respectively]) that did not produce aflatoxin (non-aflatoxigenic isolates) were selected from the collection for use in the present study. For purposes of comparison, two aflatoxigenic (E1 and E8 [GenBank HM_003463 and HM_003470, respectively])) and two non-aflatoxigenic (E2 and E9 [GenBank HM_003464 and HM_003471, respectively]) environmental isolates from the same collection were also studied.

### Culture conditions

Each fungal strain was first subcultured onto slopes of Sabouraud glucose neopeptone agar (SDA; Hi-Media, Mumbai, India) and incubated at 25–30 °C for 72 h for growth and sporulation. Conidia were harvested in physiologic saline containing 0.04% Tween-80 (Hi-Media) and suspensions of conidia were prepared to contain approximately 1×10^5^ colony-forming units (CFU)/ml. One ml of each conidial suspension was then inoculated into 150 ml of sterile glucose-salt medium [16] and incubated at 25–30 °C. The mycelia and the culture filtrates were collected from 7 day-old cultures. The mycelia were frozen and further processed for extraction of RNA, while culture filtrates were screened for presence of aflatoxin.

### RT PCR-analysis

#### Extraction of total RNA from mycelia

Total RNA was extracted from 100 mg of mycelia by using TRIZOL reagent (Sigma-Aldrich, St Louis, MO). The purity and integrity of the isolated RNA were determined by spectrophotometry and agarose gel electrophoresis.

#### cDNA synthesis and PCR amplification

Total RNA was used as the template to generate first strand cDNA in a 20 µl reaction volume as follows: 2 µg of total RNA were added to 1 µl of 10 mM dNTPs and 2 µl of 100 µM oligo dTs, made up to 10 µl with RNase-free water, heated at 70 °C for 10 min and added to a reaction mix containing 2 µl of 10× reverse transcriptase buffer, 1 µl of Moloney murine leukemia virus reverse transcriptase (M-MLV RT) enzyme (Promega, Madison, WI) and RNase-free water. The reaction mixture was incubated at 37 °C for 60 min and terminated at 95 °C for 5 min. PCR amplication of the cDNAs of the genes being studied (*afl*R, *afl*J, *nor-1, pks*A) and of a `housekeeping’ gene (β-tubulin [*TUB*]) was performed with a total reaction volume of 50 µl consisting of PCR buffer (1×), 0.2 mM each of dATP, dGTP, dCTP and dTTP, 0.5 µM of each primer (Table 1) and 1.5 µl of Taq DNA polymerase. After initial denaturation at 95 °C for 15 min, 30 cycles of amplification (denaturation at 95 °C for 30 s, annealing at 50 °C for 1 min, and extension at 72 °C for 1 min) and a final extension at 72 °C for 2 min were performed in a thermocycler (Eppendorf, Hamburg, Germany). The concentration of the template and the number of cycles were optimized to ensure linearity of the response and to avoid saturation of the reaction.

**Table 1:** Primer sequences and the expected product size of the genes studied.

**Serial number**	**Genes**	**Primer sequence**	**PCR product size (bp)**
1	*afl-R*	Forward primer 5′-CAACTCGGCGACCATCAGAG-3′	514
		Reverse primer 5′-GGGAAGAGGTGGGTCAGTGT-3′	
2	aflJ	Forward primer 5′-ATAAAGTCAGCGGCGTGGTG-3′	307
		Reverse primer 5′-ATGACCGGCACCTTAGCAGT-3′	
3	*pksA*	Forward primer 5′-TTCTGCATGGGTTCCTTGGC-3′	395
		Reverse primer 5′-CCATTGTGGGCCGGTAAACA-3′	
4	*nor-1*	Forward primer 5′-GGGATAGACCGCCTGAGGAG-3′	168
		Reverse primer 5′-CTTCAGCGACGGTTAGTGCC-3′	
5	*TUB*	Forward primer 5′-GCCGCTTTTTGACTTGCTCC-3′	231
		Reverse primer 5′-ACTGATTGCCGATACGCTGG-3	

On completion of the PCR reaction, 10 µl of each PCR product were subjected to electrophoresis in a 2% agarose gel containing ethidium bromide. Following electrophoresis, bands corresponding to transcripts of the study genes and the reference gene, *TUB*, were noted. The gel was photographed using a DS-34 type Polaroid camera and the bands were scanned by an imaging densitometer (Model GS-670; Bio-Rad' Hercules, CA); the intensity of each band was analyzed by Quantity One software (Bio-Rad). The relative expression level of each study gene was calculated as the ratio of the densitometric reading of the study gene transcript to that of *TUB*. Experiments were performed in replicates.

### Assessment of aflatoxin production in culture

#### Preparation of culture filtrate

Seven day-old broth cultures of the fungal isolates were successively filtered (Whatman No. 541 and Whatman No. 1 filter paper; Sigma Chemical Co., St. Louis, MO) and then centrifuged at 17,000× g for 30 min at 4 °C in a cooling centrifuge (Heraeus, Hanau, Germany) to yield a supernatant; this was then filtered through a 0.45 μm pore size membrane filter (Millipore, Bangalore, India) to remove any contaminating material, including fungal conidia and bacteria. The culture filtrate thus prepared was screened for the production of aflatoxin.

#### Screening for aflatoxin production

This was done by thin-layer chromatography (TLC) using a standard method [17] with some modifications. Each culture filtrate was, in succession, extracted with acetone, filtered (Whatman No. 1), extracted with chloroform in a separating funnel for 3 min, filtered, passed through anhydrous sodium sulfate, and concentrated at 60 °C to near dryness. The residue was re-suspended in chloroform and spotted in duplicate on 20×20 cm TLC silica gel plates (Merck, Darmstadt, Germany), which were developed in chloroform: methanol (98:2). Aflatoxin spots were visualized under ultraviolet light at 365 nm. Standard aflatoxin B1 (Sigma Aldrich) was used for comparison in each run. All experiments were performed at least twice.

#### Quantification of aflatoxin

Aflatoxin detected by the screening process was quantified by the method of Nabney and Nesbitt [18]. The silica gel containing the aflatoxin band was scraped off from the TLC plate and extracted with cold methanol for 3 min. The methanol was then filtered off and the silica gel was washed 5 times with fresh methanol, the combined methanolic filtrate being brought upto 5 ml; the ultraviolet absorption spectrum of the methanolic solution was then recorded. The difference between the optical density of methanolic filtrate at 363 nm and that at 420 nm was determined. This difference was then divided by the extinction coefficient (19,800) of AFB1, and the resulting figure was multiplied by the molecular weight of AFB1 (312) to obtain the concentration of aflatoxin.

### Statistical analysis

The relative expression levels of the genes studied (levels relative to those of the house- keeping gene *TUB*) are presented in Table 2 as mean±standard deviation of multiple readings made on 7 day-old fungal cultures. The statistical significance of differences between different groupings of the fungal isolates for each gene studied was determined by one-way ANOVA (ANOVA); where significant differences were noted, intergroup comparisons (between two groups) were performed by post-hoc testing using Tukey’s HSD (Statistical Package for Social Sciences-SPSS version 16.0; IBM Corp, Armonk, NY). The results of the statistical analysis for *aflR*, *aflJ*, *nor-1* and *pksA* are presented in Appendix 1.

**Table 2:** Mean relative expression levels of selected regulatory and structural aflatoxin biosynthetic pathway genes in 7 day-old corneal and environmental isolates of *Aspergillus flavus*.

Isolates of Aspergillus flavus*	Mean (±SD) relative expression levels** of aflatoxin biosynthetic pathway genes in *Aspergillus flavus*			
	*aflR*	*aflJ*	*nor-1*	*pksA*
Corneal isolate C1	0.9782±0.0315	1.0804±0.0890	0.8649±0.0310	0.9017±0.0364
Corneal isolate C4	1.0986± 0.0771	1.1786±0.0875	0.8661±0.0310	0.9344±0.0427
Corneal isolate C2	—–	1.0386±0.0600	0.6397±0.0454	0.6797±0.0348
Corneal isolate C7	0.5691±0.0757	0.6395±0.0720	0.6596±0.0315	0.6711±0.0422
Environmental isolate E1	0.9495±0.0812	1.0381±0.0955	0.8511±0.0356	0.8683±0.0334
Environmental isolate E8	0.9520±0.0516	1.0671±0.0921	0.8593±0.0381	0.8749±0.0401
Environmental isolate E2	0.5271±0.0722	0.6884±0.0841	0.6235±0.0275	0.6598±0.0313
Environmental isolate E9	0.5449±0.0875	0.5857±0.0867	0.6335±0.0299	0.6758±0.0377

*Isolates C1, C4, E1, and E8 were aflatoxigenic; isolates C2,C7, E2, and E9 were non-aflatoxigenic. **Values are expressed as mean±standard deviation of 3 readings taken from 7 day-old cultures of each fungal isolate; the mean expression levels were determined relative to that of the reference gene (*TUB*).

An attempt was also made to determine whether there were significant correlations between the quantum of aflatoxin produced and the relative expression level of each gene (Table 3); this was done by Pearson’s rank correlation (Graphpad Instat 3.10; Graphpad Software Inc., San Diego, CA).

**Table 3:** Correlation between aflatoxin production and relative expression of the regulatory (*aflR* and *aflJ*) and structural (*nor-1* and *pksA)* aflatoxin biosynthetic pathway genes.

**Fungal strains of *A. flavus***	**Gene**	Relative gene expression levels*	Quantum of aflatoxin production**	**Pearsons correlation coefficient**	**p value**	**significance**
Corneal aflatoxigenic	*aflR*	1.0384±0.0844	305.63±96.6	0.856	0.030	Significant
	*aflJ*	1.1295±0.0955		0.679	0.138	Not significant
	*nor-1*	0.9181±0.0398		0.644	0.167	Not significant
	*pksA*	0.8805±0.0325		0.576	0.232	Not significant
Environmental aflatoxigenic	*aflR*	0.9508±0.0609	210.16±11.34	0.844	0.035	Significant
	*aflJ*	1.0526±0.0854		0.946	0.004	Significant
	*nor-1*	0.8552±0.0332		0.958	0.003	Significant
	*pksA*	0.8716±0.03343		0.964	0.002	Significant
All aflatoxigenic strains	*aflR*	0.9946±0.0838	257.898±82.38	0.829	0.001	Significant
	*aflJ*	1.0911±0.0952		0.671	0.017	Significant
	*nor-1*	0.8679±0.0340		0.622	0.031	Significant
	*pksA*	0.8948±0.0426		0.678	0.015	Significant

*Values are mean±SD of three observations made on 7 day old cultures. Levels are expressed relative to those of the house-keeping gene *TUB*. **Values are mean±SD of the aflatoxin production by 7-day old cultures of aflatoxigenic clinical and environmental strains.

## Results

**Figure 1:**
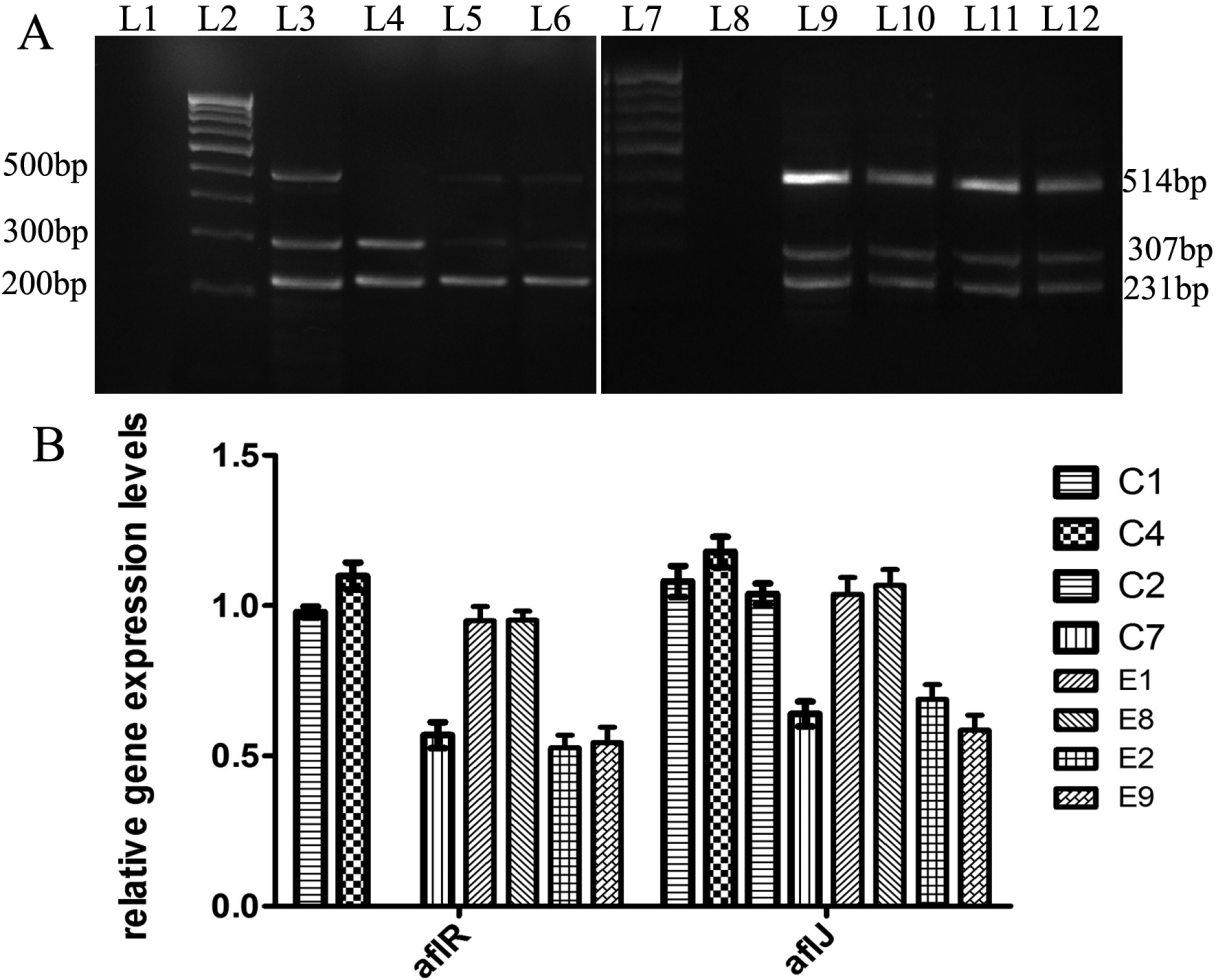
mRNA transcripts of the *β-tubulin* (231 bp), *aflR* (514 bp) and *aflJ* (307 bp) generated by RT–PCR from the corneal and environmental isolates of *Aspergillus flavus.* Ten µl of each amplified product were loaded on a 2% agarose gel. **A**: Lanes L1 and L8 were loaded with controls for the PCR reaction. Lanes L2 and L7 were loaded with a 100bp DNA marker. Lanes L3 to L6 were loaded with RT–PCR products of *aflR, aflJ* and β-tubulin genes from 7-day old cultures of corneal *A. flavus* isolates C1, C2, C4 and C7, respectively. Lanes L9 to L12 were loaded with RT–PCR products of the *aflR, aflJ* and *β-tubulin* genes from 7-day old cultures of environmental *A. flavus* isolates E1, E4, E8 and E9, respectively. **B**: Graphical representation of the relative gene expression levels of the regulatory genes *aflR* and *aflJ* of the corneal and environmental isolates of *Aspergillus flavus*.

**Figure 2:**
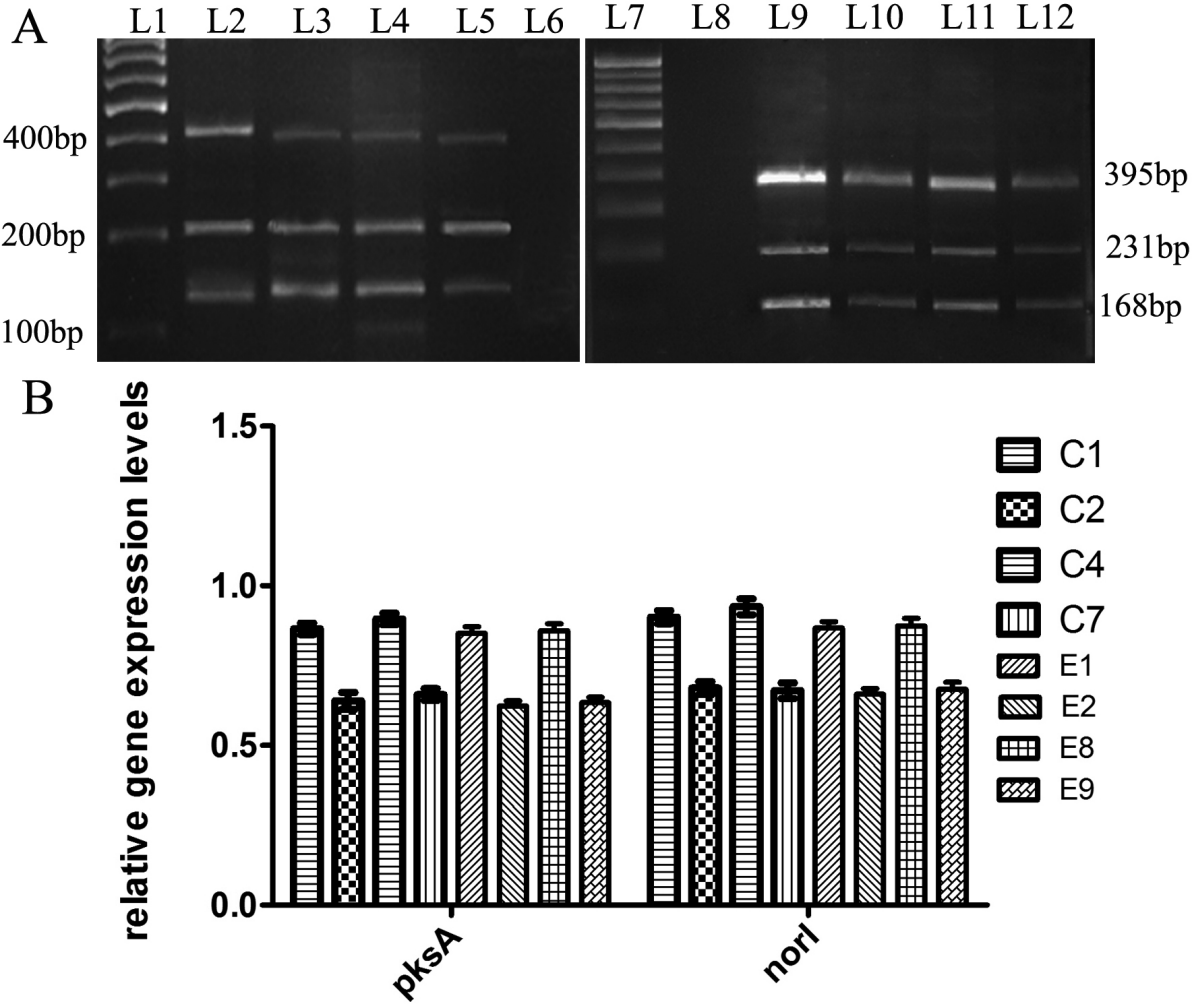
mRNA transcripts of the *β-tubulin* (231 bp), *nor-1 (168 bp)* and *pksA(395 bp)* generated by RT–PCR from the corneal and environmental isolates of *Aspergillus flavus.* Ten µl of each amplified product were loaded on a 2% agarose gel. A: Lanes L1 and L7 were loaded with a 100 bp DNA marker. Lanes L2 to L5 were loaded with RT–PCR products of *nor-1, pksA* and β-tubulin genes from 7-day old cultures of corneal *A. flavus* isolates C1, C2, C4, and C7, respectively. Lanes L6 and L8 were loaded with controls for the PCR reaction. Lanes L9 to L12 were loaded with RT–PCR products of the *nor-1, pksA* and *β-tubulin* genes from 7-day old cultures of environmental *A. flavus* isolates E1, E4, E8, and E9, respectively. B: Graphical representation of the relative gene expression levels of the regulatory genes *nor-1* and *pksA* of the corneal and environmental isolates of *Aspergillus flavus*.

**Figure 3:**
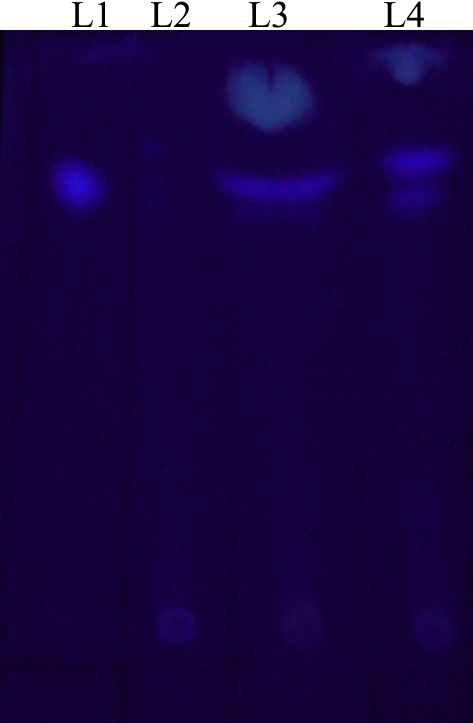
Thin layer chromatogram of culture filtrates of two corneal and two environmental isolates of *Aspergillus flavus*. L1: standard aflatoxin B1; L2: Aflatoxin B1 was not detected in the extract of the culture filtrate of corneal isolate C2; L3: Aflatoxin B1 was detected in the extract of the culture filtrate of corneal isolate C1. L4: Aflatoxin B1 was detected in the extract of the culture filtrate of environmental isolate E1.

RT–PCR (Figure 1 and Figure 2), detected transcriptional activation (expression) of the *aflJ*, *nor-1*, and *pksA* genes in all four corneal isolates of *A. flavus*, and expression of *aflR* in three of the four isolates (Table 2); a 7-day-old culture of C2 did not express *aflR*, but the transcripts of the *aflJ*, *nor-1*, and *pksA* genes were still detected in this isolate (Table 2). The mean expression levels of *aflR, nor-1*, and *pksA* were significantly higher in the aflatoxigenic corneal isolates (C1, C4) than in the non-aflatoxigenic corneal isolates (C2, C7); the mean expression levels of *aflJ* in the corneal aflatoxigenic isolates C1 and C4 were significantly higher than that in the non-aflatoxigenic corneal isolate C7 (Appendix 1, a-d). Screening for aflatoxin production by TLC (Figure 3), followed by quantification by spectrophotometry, confirmed the production of AFB1 by corneal isolates C1 and C4, but not by C2 and C7. In the corneal aflatoxigenic isolates, a significant positive correlation was noted between mean aflatoxin production and the mean relative expression level of *aflR* (p=0.030) only (Table 3).

Interestingly, the gene expression results obtained with the four environmental isolates tested as a comparison were mostly similar to those obtained with the corneal isolates (Figure 1 and Figure 2; Table 2 and Table 3), with some exceptions. In contrast to the corneal isolates, all four environmental isolates were found to express all four genes (Figure 1 and Figure 2; Table 2).

Similar to the corneal isolates, the mean expression levels of *aflR, aflJ, nor-1*, and *pksA* were significantly higher in aflatoxigenic environmental isolates (E1, E8) than the levels in non-aflatoxigenic environmental isolates (E2, E9; Appendix 1, a-d). Among the environmental isolates, aflatoxin B1was found to be produced by E1 and E8, but not by E2 and E9 (Figure 3). Significant (p=0.002 to p=0.035) positive correlations emerged between mean aflatoxin production and mean relative expression levels of all four genes studied in the environmental aflatoxigenic *A. flavus* isolates (Table 3).

There were no significant differences between aflatoxigenic corneal and aflatoxigenic environmental isolates in mean expression levels of all four genes, or between non-aflatoxigenic corneal and non- aflatoxigenic environmental isolates in mean expression levels of *nor-1* and *pksA* (Appendix 1, a-d).

## Discussion

The expression of aflatoxin biosynthetic pathway genes has been studied in isolates of *A. flavus* from the field [13], but not in those from clinical sources. Our investigation is possibly the first to examine the expression of regulatory and structural aflatoxin biosynthesis genes in *A. flavus* isolates from patients with keratitis.

Accinelli et al. [13] reported that approximately 60% of *A. flavus* isolates from soil were aflatoxigenic (had the potential to produce aflatoxins). We have previously reported that AFB1 was detected in 80% of growth samples from clinical isolates of *A. flavus* (from patients with keratitis) but in only 40% of growth samples from environmental *A. flavus* isolates [14]. We sought to investigate whether the observed differences in aflatoxin-producing ability could be explained by differences in expression of aflatoxin biosynthesis genes between *A. flavus* isolates from patients with keratitis and those from the environment. We decided to test out this hypothesis by quantifying relative expression levels of the genes in these isolates. We used RT–PCR for this purpose since, although only a semi-quantitative method, it has been found accurate in differentiating between aflatoxigenic and non-aflatoxigenic isolates of *A. flavus* in agricultural commodities [19], and has also been used by other workers [13,20] to study aflatoxin biosynthetic genes in *A. flavus.* Thin layer chromatography (TLC) was used to quantify aflatoxin production by the isolates since this is the official method of the Association of Agricultural Chemists (AOAC) and can identify and quantify aflatoxins at levels as low as 1 ng/g. In fact, this method is the basic technology for verification of newer techniques of aflatoxin detection; in other words, this technique can be considered as the ‘gold standard’.

Accinelli et al. [13] sought to elucidate the potential of soil *A. flavus* to produce aflatoxins by detecting, by RT–PCR, transcription of five aflatoxin biosynthesis genes, namely, *aflD, aflG, aflP, aflR*, and *aflS*. We selected four genes for study, namely, two regulatory genes, *aflR* and *aflJ*, and two structural genes, *nor-I* and *pksA*, based on their role in aflatoxin biosynthesis. *aflR* encodes a putative 47 kDa protein, aflR, which contains a zinc cluster-DNA binding motif that is required for the transcriptional activation of all the characterized aflatoxin pathway genes [21,22]. The specific interaction between aflJ and aflR suggests that *aflJ* is directly involved in the regulation of transcription of structural genes for enzymes in the aflatoxin pathway [23]. The transcriptional activity of *nor-1* and *pksA* has been found to increase dramatically during aflatoxin accumulation in culture [24-26]. In the study by Chang and Hua [27], non-aflatoxigenic isolates were found to be genetically different from an aflatoxin-producing isolate due to the presence of polymorphism in *pksA*, which resulted in the production of a defective polyketide synthase.

In the current study, we observed significantly higher mean (relative) expression levels of *aflR* in aflatoxigenic isolates of *A. flavus* (both corneal and environmental) than in non-aflatoxigenic isolates of *A. flavus* (both corneal and environmental; Appendix 1, a); we also noted a significant positive correlation between expression of *aflR* and mean aflatoxin production in corneal and environmental aflatoxigenic isolates (Table 3). Interestingly, although *aflR* was not expressed at all by one non-aflatoxigenic corneal isolate (C2), *aflJ*, *pksA*, and *nor-1* were still expressed by this isolate; a possible explanation for this is that under normal conditions, expression of structural genes (including *nor-1* and *pksA*) requires specific factors (for example, sugars) that induce transcription [22].

Disruption of *aflJ* in *A. flavus* has been found to result in failure to produce any aflatoxin pathway metabolites without, however, interfering with the generation of transcripts for many of the aflatoxin structural genes [28]. This latter observation may explain our finding that, although the expression pattern of *aflJ* differed in some aspects from that of *aflR*, the expression patterns of *nor-1* and *pksA* still mirrored that of *aflR* in the isolates of *A. flavus.*

We observed significant positive correlations between the expression of all four study genes and mean aflatoxin production in the environmental aflatoxigenic isolates, and between mean expression of *aflR* and mean aflatoxin production in corneal aflatoxigenic isolates. This suggests that enhanced expression of all four study genes in the environmental aflatoxigenic isolates manifested in increased aflatoxin production whereas enhanced expression of *aflR* alone in corneal aflatoxigenic isolates translated into increased aflatoxin production. While the exact significance of this finding is uncertain, it is interesting to note that our observation is consistent with those made in studies on *A. flavus* strains isolated from corn grains collected from fields in Italy [20] and from soils in Canada [13]. In the study by Degola et al. [20] five *A. flavus* strains expressed all the study genes (*aflR*, *aflS*, *aflO*, *aflQ*, and *aflD*), and a good correlation between gene detection, gene expression and aflatoxin production was observed in all these strains except one (aflatoxin non-producing) strain. In the study by Accinelli et al. [13] the overall profile of the genes *(aflD, aflG, aflP, aflR, aflS)* in the soil, with some genes being expressed and others not expressed, was not generally related to concentration of AFB1; however, the soil in which there was a complete positive gene expression profile also revealed the highest concentration of AFB1 [13]. Thus, the consequences of aflatoxin biosynthetic gene expression in the cornea may differ from that in the environment.

The results of our study suggest that expression of aflatoxin biosynthetic genes in *A. flavus* occurs both in strains that produce aflatoxin and those that do not. Possibly, there is a threshold level for expression of the relevant genes. Where genes are expressed above this threshold, aflatoxin production is induced. This would explain our finding of significantly higher expression levels of aflatoxin biosynthetic genes in aflatoxigenic isolates than in non-aflatoxigenic isolates. Interestingly, Kale et al. [29] observed that *A. parasiticus* variants lacking the *sec* gene (sec^-^ strains) exhibited altered phenotypes and an inability to produce aflatoxin intermediates, compared to *sec*+ strains; however, the *sec*^-^ variants still produced transcripts of aflatoxin genes, such as *aflR*, *aflD* and *aflP*.

When Chang et al. [30] compared expression of aflatoxin biosynthetic genes in three aflatoxigenic parental *A. flavus* strains with that in three progeny nonaflatoxigenic strains, they observed that none of the genes studied were significantly different by their defined parameters; this led them to suggest that loss of aflatoxin production in the progeny strains was not caused directly by altered expression levels of the aflatoxin biosynthetic genes, but, possibly, indirectly by nutritional and physiologic factors that induced changes in primary metabolism, cell cycle and differentiation. Production of aflatoxin by toxigenic *Aspergilli* is known to be influenced by environmental and nutritional factors, such as temperature, pH, carbon and nitrogen source, stress factors, lipids and certain metallic salts [31].

Aflatoxin is produced by *Aspergillus flavus* and *Aspergillus parasiticus*. However, the relevance of aflatoxin as a virulence factor in clinical fungal keratitis is uncertain. The comparatively higher frequency of reports of *A.flavus* keratitis, compared to the few reports of *A.parasiticus* keratitis may reflect additional virulence factors in *A.flavus* such as invasive potential and relative resistance to certain antifungal agents. Although the adaptive value of aflatoxin production is not fully understood, it has been postulated that synthesis of aflatoxins may serve as a defense mechanism against oxidative stress [30]. In this context, it is interesting to note that antioxidants have been shown to reduce aflatoxin production [32] and that a positive correlation has been shown between accumulation of reactive oxygen species and production of aflatoxin by *A. parasiticus* [33,34]. The results of a series of laboratory experiments suggested that adverse environmental conditions (high temperature, low pH, and nutrient deprivation), but not competition with yeasts and filamentous fungi, helped to maintain aflatoxigenicity over successive generations during serial transfers [35].

A complex interaction of temperature, water activity, incubation period, and substrate has been found to influence the relative concentrations of aflatoxins produced by *A. flavus* [36]. In this context, the normal corneal temperature of 33 °C to 34 °C [37,38] may stimulate synthesis of aflatoxin. Similarly, a low pH in corneal tissue infected by *A. flavus* may favor aflatoxin production. Our previous observation [14] that significantly higher percentages of corneal *A. flavus* isolates than environmental *A. flavus* isolates were aflatoxigenic may have been because conditions in the cornea possibly influence expression of aflatoxin biosynthesis genes to a greater extent than do conditions in the external (natural) environment.

Thus, corneal tissue infected by *A. flavus* may provide a favorable setting for increased expression of regulatory (*aflR* and *aflJ*) and then structural (*nor-I* and *pksA*) aflatoxin biosynthetic genes, therein modulating aflatoxin production by the fungus in the cornea; this may augment the virulence of the fungal isolate infecting the cornea. This effect possibly persists even after the fungus is isolated in culture from corneal scrape material, and subcultured to culture media. These hypotheses need to be confirmed by detection of aflatoxin in corneal tissue infected by aflatoxigenic strains of *A. flavus* and by demonstrating increased expression of aflatoxin biosynthesis genes and increased production of aflatoxin following transfer of non-aflatoxigenic isolates of *A. flavus* from the natural environment to corneal tissue.

## Appendix 1. The results of the statistical analysis for *aflR*, *aflJ*, *nor-1* and *pksA*.

**a**: Relative expression of the regulatory gene *aflR *by isolates of *Aspergillus flavus*. **b**: Relative expression of the regulatory gene *aflJ *by isolates of *Aspergillus flavus*. **c**: Relative expression of the structural gene *norI *by isolates of *Aspergillus flavus*. **d**: Relative expression of the structural gene *pksA *by isolates of *Aspergillus flavus*. To access the data, click or select the words “Appendix 1.” This will initiate the download of a compressed (pdf) archive that contains the file.
